# Comparative phenotypic and functional analysis of migratory dendritic cell subsets from human oral mucosa and skin

**DOI:** 10.1371/journal.pone.0180333

**Published:** 2017-07-13

**Authors:** Ilona Jennifer Kosten, Rieneke van de Ven, Maria Thon, Susan Gibbs, Tanja D. de Gruijl

**Affiliations:** 1 Department of Dermatology, VU University Medical Center, Amsterdam, the Netherlands; 2 Department of Medical Oncology, VU University Medical Center, Amsterdam, the Netherlands; 3 Department of Oral Cell Biology, Academic Center for Dentistry Amsterdam, University of Amsterdam and VU University, Amsterdam, the Netherlands; Purdue University, UNITED STATES

## Abstract

Antigen exposure to oral mucosa is generally thought to lead to immune tolerance induction. However, very little is known about the subset composition and function of dendritic cells (DC) migrating from human oral mucosa. Here we show that migratory DC from healthy human gingival explants consist of the same phenotypic subsets in the same frequency distribution as DC migrating from human skin. The gingival CD1a^+^ Langerhans cell and interstitial DC subsets lacked CXCR4 expression in contrast to their cutaneous counterparts, pointing to different migration mechanisms, consistent with previous observations in constructed skin and gingival equivalents. Remarkably, without any exogenous conditioning, gingival explants released higher levels of inflammatory cytokines than human skin explants, resulting in higher DC migration rates and a superior ability of migrated DC to prime allogeneic T cells and to induce type-1 effector T cell differentiation. From these observations we conclude that rather than an intrinsic ability to induce T cell tolerance, DC migrating from oral mucosa may have a propensity to induce effector T cell immunity and maintain a high state of alert against possible pathogenic intruders in the steady state. These findings may have implications for oral immunization strategies.

## Introduction

Dendritic cells (DC) that are located in epithelia at the interface with the outside environment form a primary barrier of defence against pathogenic intruders. They are powerful antigen presenting cells (APC), linking innate to adaptive immunity. As such they perform a delicate balancing act, maintaining immune tolerance under steady-state conditions but also inducing T cell immunity when needed. During homeostasis, migrating immature DC from peripheral tissues take up antigen but do not acquire the capacity to promote functional T cell-mediated immune responses [1,2]. However, upon their recognition through specialized receptors of pathogen- or damage-associated molecular patterns (PAMPs and DAMPs respectively), they are activated, migrate to the draining Lymph Nodes (LNs), and mature into potent immune stimulators that can drive T cell induction, expansion and differentiation [3–5].

In human skin, at least five major DC subsets have been described, primarily distinguishable by their differential expression of CD1a and CD14, i.e. epidermal Langerhans cells, characterized by high levels of CD1a and Langerin expression, and four interstitial dermal DC (DDC) subsets, including CD1a^+^ and CD14^+^ DDC [6]. We previously showed that the frequency distribution between these migrating subsets and thereby the eventual T cell activation outcome, depended on the activating versus regulatory cytokine balance in the skin microenvironment [6]. Under the influence of suppressive IL-10, migration of CD14^+^ DDC prevails, resulting in abortive T cell priming and regulatory T cell (Treg) induction and expansion [6]. Under pro-inflammatory conditions (e.g. high levels of GM-CSF and/or IL-4) migration of CD1a^+^ LC and DDC subsets is dominant, leading to Th1 and cytotoxic T cell (CTL) induction and expansion. Thus, the frequency distribution of migratory DC subsets from human skin determines subsequent T cell activation or tolerance induction [7,8].

The oral cavity is daily exposed to a high burden of antigens emanating from food, bacteria, viruses, fungi, and their by-products. The oral mucosa thus forms a major interface with the outside world, and its integrity and appropriate response to antigens are crucial to maintain health [9]. Like gut mucosa, oral mucosa is generally assumed to be instrumental in maintaining immune tolerance against the daily onslaught of harmless food antigens and commensal bacteria. As such, the distribution of migratory DC subsets (and consequently their net T cell skewing capacity) might be expected to differ from that in skin, where in the steady-state usually CD1a^+^ LC and DDC migration predominates with default priming of a type-1 T cell response in the allogeneic mixed leukocyte response [6]. As yet, very little is known about DC subsets in human oral mucosa. No flow cytometric analyses of migrated DC from oral mucosa explants have been reported, due to a general scarcity of available tissue. So far LC have been mainly studied, showing their presence in oral mucosa [10–12] and their superior ability to prime allogeneic T cells as compared to their skin counterparts [13]. Of note, oral LC were further shown to differ from their skin counterparts by their expression of lipopolysaccharide receptor/CD14 and the high affinity receptor for IgE (FcεRI), possibly allowing for more efficient activation by gram-negative bacteria and allergen uptake, respectively [12]. In addition to LC, DC-SIGN^+^ DC were observed in the lamina propria of oral mucosa [14].

We assessed the distribution, maturation state and functionality of human oral mucosa associated migratory DC subsets in a comparative analysis with their skin counterparts. Flowcytometric and T cell priming analyses showed a similar subset distribution and activation state among gingiva-migrated DC, but, surprisingly, also revealed their superior type-1 T cell skewing capacity. These data call for a reappraisal of the functionality of oral mucosa-associated DC subsets and shed new light on oral tolerance and immunisation.

## Materials and methods

### Tissue samples

Human adult skin was obtained from 15 healthy donors undergoing corrective breast or abdominal plastic surgery. Human adult non-inflamed gingival tissue was obtained from 15 healthy donors receiving dental implants or undergoing wisdom tooth extraction under local anaesthesia. Tissue specimens were collected after informed verbal consent, and used in an anonymous fashion in accordance with the “Code for Proper Use of Human Tissues” as formulated by the Dutch Federation of Medical Scientific Organizations (www.fmwv.nl) and following consent procedures approved by the institutional review board of the VU University medical center. The study was conducted according to the Declaration of Helsinki Principles. Skin and gingiva samples were not donor matched.

### Immunohistochemistry

For immunohistochemical staining of gingiva and skin biopsies the samples were either snap-frozen in liquid nitrogen or embedded in paraffin. Vertical 5 μm cryostat frozen sections were cut from 10 different donors, and air-dried at room temperature on SuperFrost® gold slides (Menzel GmbH & Co KG, Braunschweig, Germany). The cryostat frozen sections were fixed in acetone (VWR, Amsterdam, the Netherlands) for 10 minutes and incubated for 60 minutes with primary monoclonal antibodies directed against the different surface markers, as listed in Table 1.

**Table 1:** Monoclonal antibodies used for immunohistochemical staining.

Primary mAb	Species	Clone	Serial no.	Manufacturer
**Paraffin and cryo**				
Langerin	mouse IgG2b	12D6	NCL-Langerin	Leica
HLA-DR	mouse IgG1	TAL.1B5		Dako
DC-Sign	mouse IgG2bκ	DCN46		BD Pharmingen
CD14	mouse IgG2aκ	TÜK4		Dako
CD83	mouse IgG1	1H4b		Monosan
CD86	mouse IgG1	2331		BD Pharmingen
CD68	mouse IgG1κ	EBM11	M0718	Dako
**Paraffin only**				
CD1a	mouse IgG1	MTB1	MONX10315	Monosan
**Cryo only**				
CD1a	mouse IgG1	JPM30	NCL-CD1a-220	Leica

After washing, sections were incubated with goat anti-mouse conjugated to HRP (Envision, DakoCytomation). Subsequently slides were rinsed and incubated for 10 minutes with 3-amino-9-ethylcarbazole (Invitrogen, San Francisco, CA, USA). All sections were counter-stained with Mayer’s haematoxylin (Sigma Chemical Co., St Louis, MO, USA). Negative controls were prepared by omitting the primary antibody and substituting an isotype control antibody. The sections were embedded in Aquatex® (Merck).

The 5 μm paraffin embedded sections were deparaffinized and rehydrated in preparation for immunohistochemical analysis, carried out as previously described [15]. In brief, antigen retrieval was performed using citrate buffer. Subsequently sections were incubated O/N at RT with primary monoclonal antibodies directed against the different surface markers (see Table 1). After washing in PBS for 5 minutes, sections were incubated for another 30 minutes with human anti-mouse conjugated to HRP. After once again washing with PBS, the slides were incubated for 10 minutes with 3-amino-9-ethylcarbazole. All sections were counter-stained with haematoxylin. Negative controls were prepared by omitting the primary antibody and substituting an isotype control antibody. The sections were embedded in Aquatex®. The number of cells was quantified with the aid of Nis Elements AR version 3.2 Software (Nikon Instruments Europe B.V., Amstelveen, the Netherlands).

### Quantitation of cell populations

Assessment and quantitation of cell numbers after immunostaining were performed by two independent investigators. The number of positively stained cells in the epidermis, dermis, mucosal epithelium and subjacent lamina propria, were assessed for each sample per 100 μm^2^ tissue at 200x magnification, with an ocular objective of 20×, an eyepiece of 10× and a tube factor 1. The average number of stained cells was then expressed per 100 μm2 of tissue examined.

### Skin and gingival explant preparation and culture

An exact punch biopsy of 6 mm diameter and 3 mm deep was taken from skin or gingiva, consisting of eptihelium and underlying dermis or lamina propria, respectively. Of note, the full-thickness skin and gingiva explants were of equivalent depths to ensure valid comparisons of numbers of emigrated DC and secreted cytokines. Explants were then placed in 1 ml culture medium (i.e. minimal essential media (MEM)-alpha (Gibco, Grand Island, NY) supplemented with 20% v/v heat inactivated fetal calf serum (Hyclone Laboratories, Logan, UT), 1% penicillin-streptomycin, 2mM L-glutamine (Invitrogen), 50μM 2-ME (Merck, Whitehouse Station, NY) allowing the cells to migrate from the biopsies for 48 h, after which they were harvested and analyzed by flowcytometry. The skin and gingiva explants were discarded. Conditioned medium and migrated cells were harvested at this time and used for further analyses by flowcytometry, a mixed lymphocyte reaction (MLR) or Cytometric Bead Assay (CBA).

### Inflammatory Cytometric Bead Assay (CBA)

Conditioned medium that was collected from the explant cultures after 48 h was analysed for secreted IL-8, IL-1β, IL-6, IL-10, TNFα and IL-12p70 using the inflammatory CBA kit (BD, San Jose, CA) according to the manufacturer’s instructions and using CBA analysis software (BD Biosciences).

### Flow cytometry

Phenotypic analyses were performed by flow cytometry. Skin or gingiva emigrated cells were washed and resuspended in PBS supplemented with 0.1% BSA and 0.1% NaN3 (PBA) and incubated for 30 min. at 4°C in the presence of appropriate dilutions in PBA of FITC, PE, PerCP or APC fluorochrome-conjugated specific mAbs to CD11c, CD14, CD1a, CCR6, CCR7, CXCR4, CD163, CD3, CD4, CD8, CD19 (BD, San Jose, CA), Langerin, CD86 or CD83 (Beckman Coulter Immunotech), or corresponding isotype-matched control mAbs (BD, San Jose, CA) as described previously [6]. The cells were subsequently analyzed, using a FACSCalibur and Cellquest-Pro FACS analysis software (BD, San Jose, CA).

### Allogeneic T cell differentiation induction

Peripheral blood mononuclear cells (PBMC) were isolated by Lymphoprep (Pharma AS, Oslo, Norway) gradient centrifugation from a buffy coat (Sanquin Blood supplies, Amsterdam, the Netherlands) and the monocytes were depleted after 2 h plastic adherence, leaving peripheral blood lymhocytes (PBL). From the explants, the migrated cells were harvested and the DC population was counted. Three thousand DC (pooled per condition) were co-seeded with 30,000 PBL in a 96-well round bottom plate, in duplicate for 6 days in IMDM supplemented with 10% Human Pooled Serum (Sanquin Blood Supply, Amsterdam, the Netherlands), 100IU/ml sodium penicillin (Yamanouchi Pharma), 100IU/ml streptomycin sulphate (Radiumfarma-Fisiopharma), 2 mM L-glutamine (Invitrogen), and 0.01 mM 2-ME (Merck). On day 6 the supernatants were collected for the simultaneous flowcytometric detection of IL-2, IL-4, IL-6, IL-10, TNFα, IL-17A and IFNγ, secreted by the T cells using a Th1/Th2/Th17 CBA kit (BD, San Jose, CA) following the manufacturer’s instructions and using CBA analysis software (BD Biosciences).

### Statistical analysis

Differences between gingiva- and skin-resident DC counts were analysed by Welch's unequal variances t-test and migrated DC subsets and cytokine release levels by the unpaired student t-test or Mann-Whitney U test; differences were considered significant when p<0.05.

## Results

### DC density and localization in skin vs gingiva

**Fig 1:**
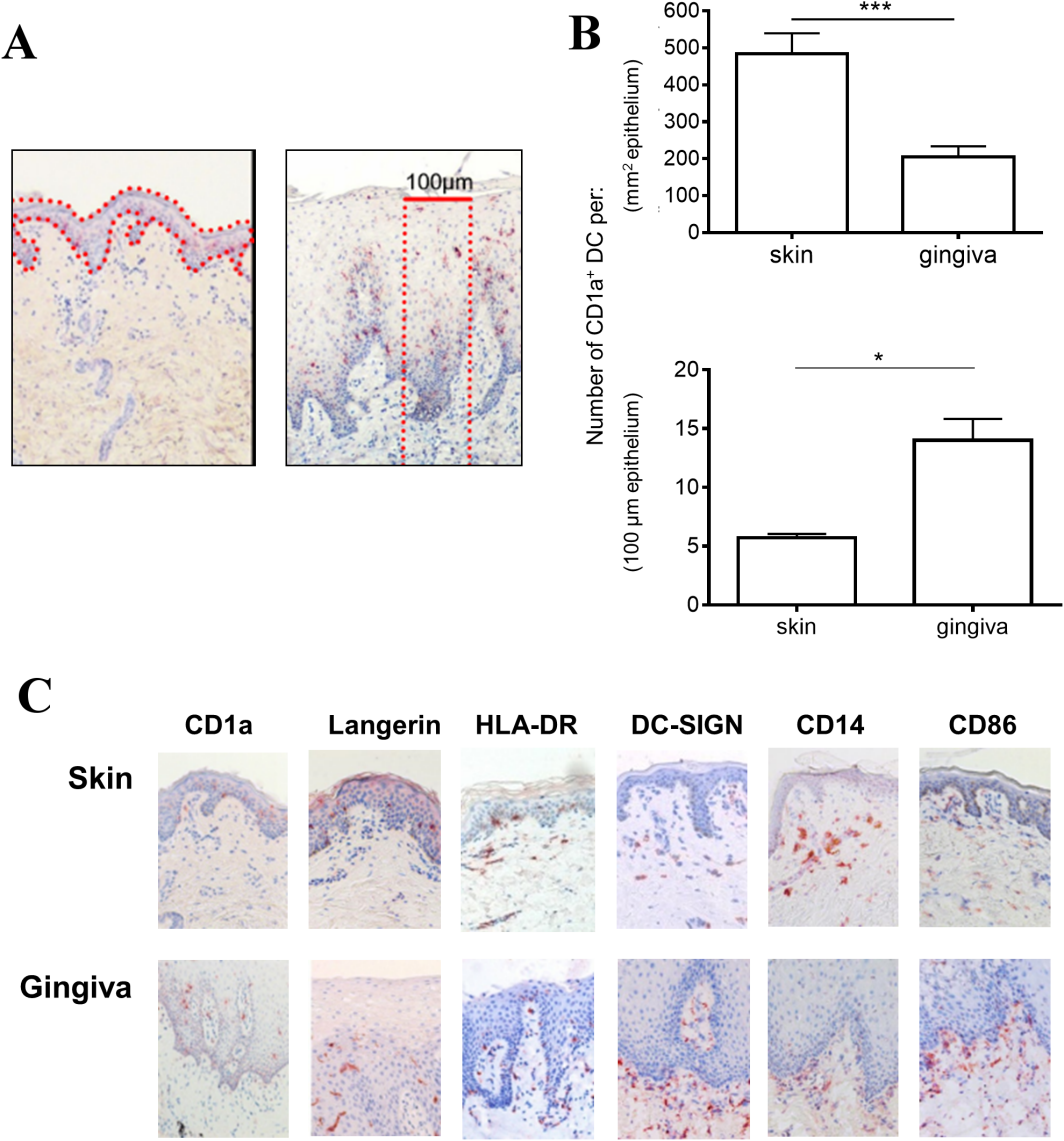
Dendritic cell (DC) marker expression, density and distribution over full-thickness human gingiva or skin. (A) CD1a staining of representative full-thickness skin (left panel) and gingiva (right panel) biopsies. Red dotted lines denote full-area epithelial surface (left panel, used for quantitation as shown in Fig 1B upper panel) and 100 μm wide full-thickness cross-section (right panel, used for quantitation as shown in Fig 1B lower panel, see also Materials and Methods). (B) Quantitation of CD1a^+^ DC according to total epithelium area (upper panel) or epithelium area in 100 μm wide full-thickness cross-section (lower panel), see areas denoted by red dotted lines in Fig 1A for the respective definitions (n = 10). The number of positively stained cells in the epidermis, dermis, mucosal epithelium and subjacent lamina propria, were assessed for each sample per 100 μm^2^ tissue. * P<0.05, ***P<0.001. (C) Representative staining of indicated additional DC maturation/differentiation markers shows distribution between epithelial and underlying connective tissue layers in skin and gingival biopsies (n = 10).

Immunohistochemical analysis of full-thickness steady-state skin and gingiva tissue revealed higher density of CD1a^+^ LC per mm^2^ epithelium in skin (Fig 1A and 1B). However, as shown in Fig 1A, the gingival stratified squamous epithelium was considerably thicker than the epidermis of the skin. Trans-epithelial assaults by e.g. pathogens, allergens or irritants, can effectively be countered by the DC encountered on the way. We therefore decided to quantify the number of LC and interstitial DC over the full-thickness (i.e. the epithelium and the underlying connective tissue layer) of skin or gingiva over a 100 μm cross-section (see Fig 1A). As shown in Fig 1B and Table 2, this resulted in an opposite result with an effectively higher density of DC per 100 μm tissue cross-section in gingiva (identified by CD1a -and Langerin staining in the epithelium). Similarly, DC-SIGN^+^ cell numbers (located in the connective tissue underlying the epithelium) were higher in gingiva, as were HLA-DR^+^ LC and interstitial DC (Fig 1C and Table 2). The CD14^+^ and CD68^+^ cell counts were comparable between the two tissue types, as were the CD83^+^ and CD86^+^ cells (all localized to the interstitial, connective tissue), although the latter two showed considerable inter-donor variation (Fig 1C and Table 2).

**Table 2:** Comparison of cell density between full-thickness skin and gingiva (per 100μm tissue cross-section).

Primary mAb	Skin*	Gingiva*	P**
**Paraffin and cryo**			
CD1a	5.71 ± 1.5	14 ± 7.5	0.0003
Langerin	4.83 ± 1.66	14.5 ± 9.3	0.0063
DC-SIGN	6.42 ± 1.84	13.78 ± 4.7	0.0013
HLA-DR	10.29 ± 1.38	18.67 ± 6.6	0.0266
CD14	6.74 ± 2.32	6.63 ± 2.91	0.9298
CD68	8.73 ± 2.37	8.76 ± 2.88	0.9854
CD83	1.45 ± 2.16	2.24 ± 1.6	0.2755
CD86	2.16 ± 2.23	0.87 ± 0.99	0.0548

*Means and standard deviations, calculated over n = 7–21

** By Welch's unequal variances t-test

### DC subset frequency distribution upon migration from skin or gingiva

**Fig 2:**
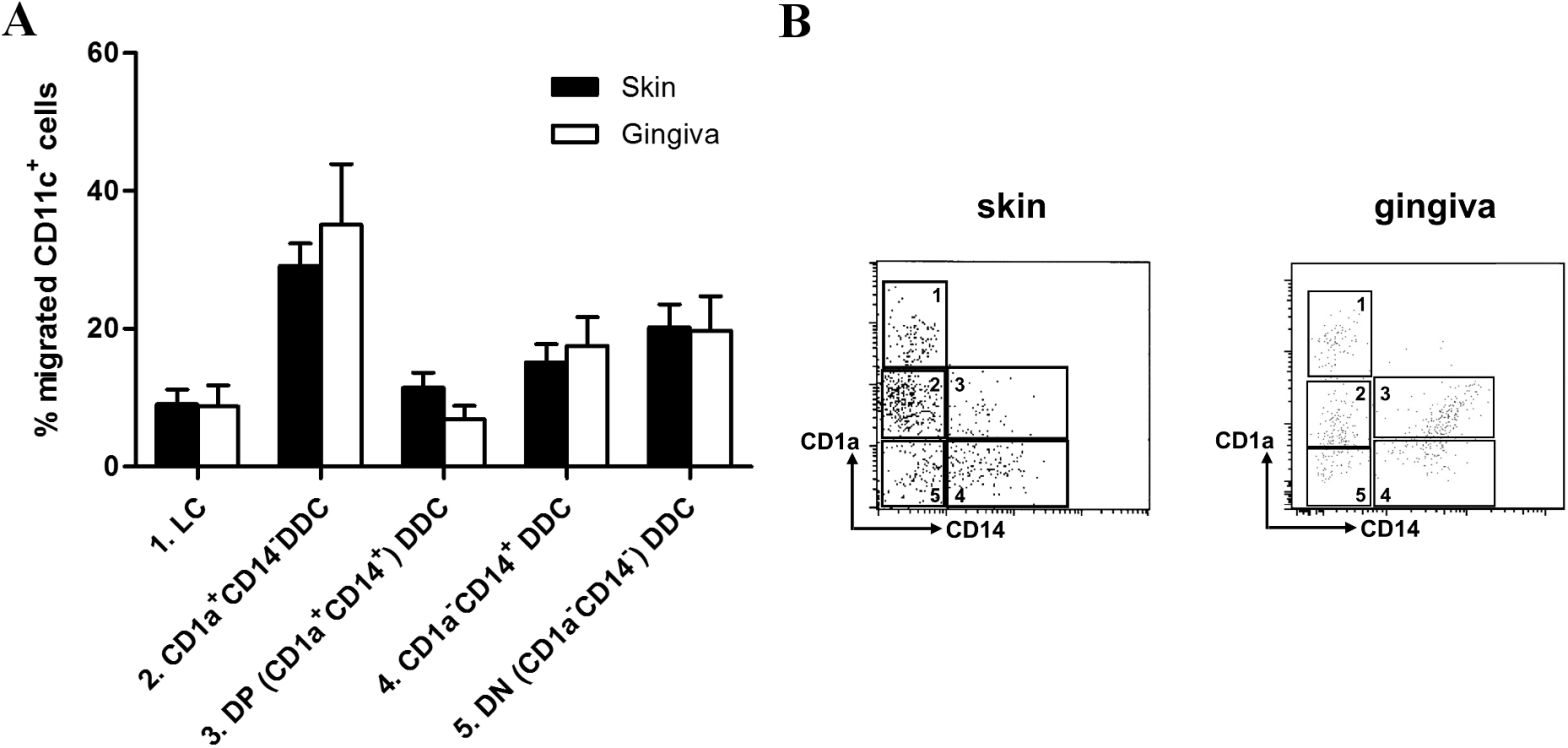
Dendritic cell (DC) subset definition and distribution. Dendritic cell (DC) subset definition and distribution according to CD1a and CD14 expression in CD11c^hi^ DC migrated from human skin or gingiva. Explants (6mm diameter) from skin and gingiva were taken and cultured floating in medium for 48h, after which they were discarded and migrated DC harvested, stained and analysed by flowcytometry. (A) Flow cytometry dot plots with gates denoting five migrated DC subsets (numbered 1 to 5) in skin and gingiva. (B) Frequency distribution of the five subsets among migrated DC (n = 9).

Full-thickness skin and gingiva explants of similar size and thickness were cultured for 2 days and crawl-out cells were subsequently harvested, counted and analyzed by flow cytometry. Of note, substantially higher numbers of migrated cells were observed for gingiva (mean 32,763 cells/explant, range 22,500–50,050, n = 4) than for skin (mean 10,937 cells/explant, range 7,590–15,300, n = 3) (P = 0.039). Gating on CD11c^hi^ cells, we distinguished five emigrated DC subsets, based on CD1a and CD14 expression, as described previously [6], i.e. 1) CD1a^hi^ LC, 2) CD1a^+^CD14^-^, 3) CD1a^+^CD14^+^ (double positive, DP), 4) CD1a^-^CD14^+^, and 5) CD1a^-^CD14^-^ (double negative, DN) interstitial DC (see Fig 2A). Of note, the frequency distribution of these subsets within the migrated cell population was equivalent between skin and gingiva (Fig 2B), with CD1a^+^CD14^-^ interstitial DC being the most frequent. The most striking (but not significant) difference was between the DP interstitial DC subsets, where skin-emigrated DC on average contained almost twice the amount of DP cells (skin: 15% vs. gingiva: 8%).

### Phenotypic profiling of gingiva- vs skin-emigrated DC subsets

**Fig 3:**
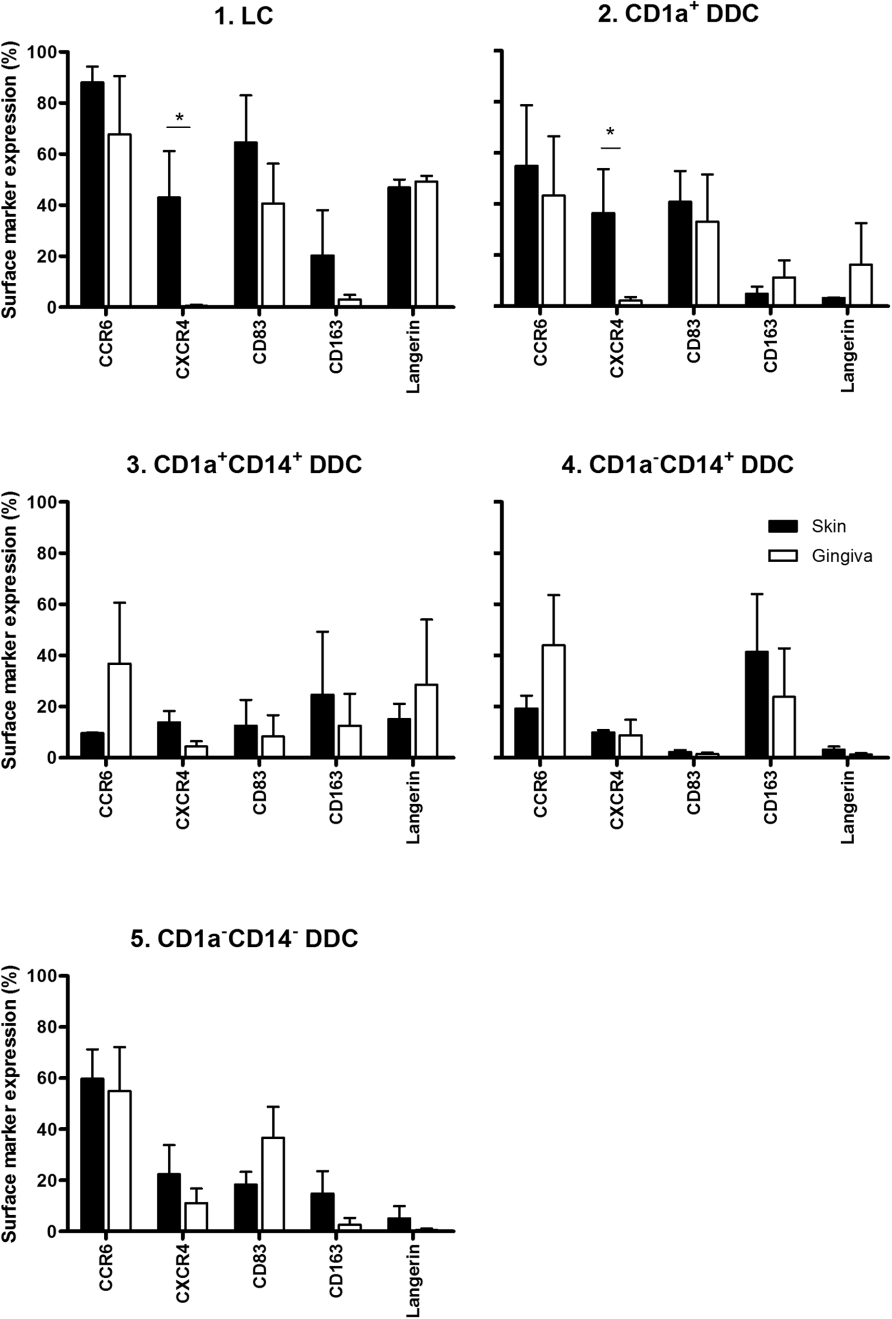
Phenotypic analysis of migratory dendritic cell (DC) subsets from skin and gingiva. Chemotaxis, maturation and differentiation-associated marker expression on DC subsets 1–5 from skin vs. gingiva, shown per indicated subset (*P<0.05, n = 4–9 for skin and gingiva). Explants (6mm diameter) from skin and gingiva were taken and cultured floating in medium for 48h, after which they were discarded and migrated DC harvested, stained and analysed by flowcytometry.

We next studied the phenotypes of the migrated DC subsets in more detail, focusing on maturation/differentiation state (CD83, Langerin, CD163) and migratory capacity (CCR6, CXCR4) (Fig 3). Of note, in general very similar expression levels of these markers were observed for each particular subset, irrespective of tissue origins. Both in skin and gingiva, CD1a^+^ subsets were more mature as judged by CD83 expression levels, consistent with our previous observations in skin [6].

As expected, highest Langerin surface levels were observed in the LC subsets from both skin and gingiva. Lower but detectable Langerin expression levels were observed on the CD1a^+^ and DP subsets from gingiva, which were notably higher than on their counterparts from skin, although not significantly so. As previously found for skin, The M2 macrophage related marker CD163 was highest on the CD14-expressing DC subsets. Expression of CCR6, generally associated with skin/epidermal homing, was very high on the CD1a^+^ and DN subsets, but, remarkably, for gingiva also on the CD14^+^ subsets. Finally, the only significant and most profound difference was found in CXCR4 surface expression levels between skin- and gingiva-emigrated LC and CD1a^+^ interstitial DC, with high expression levels on the skin-emigrated subsets and virtually no expression on gingiva-derived subsets. This finding is highly suggestive of differential epithelium-to-connective-tissue LC migration mechanisms between these tissue types[3,16,17].

### Pro-inflammatory cytokine release in gingiva and preferential type-1 T cell induction by migratory DC

**Fig 4:**
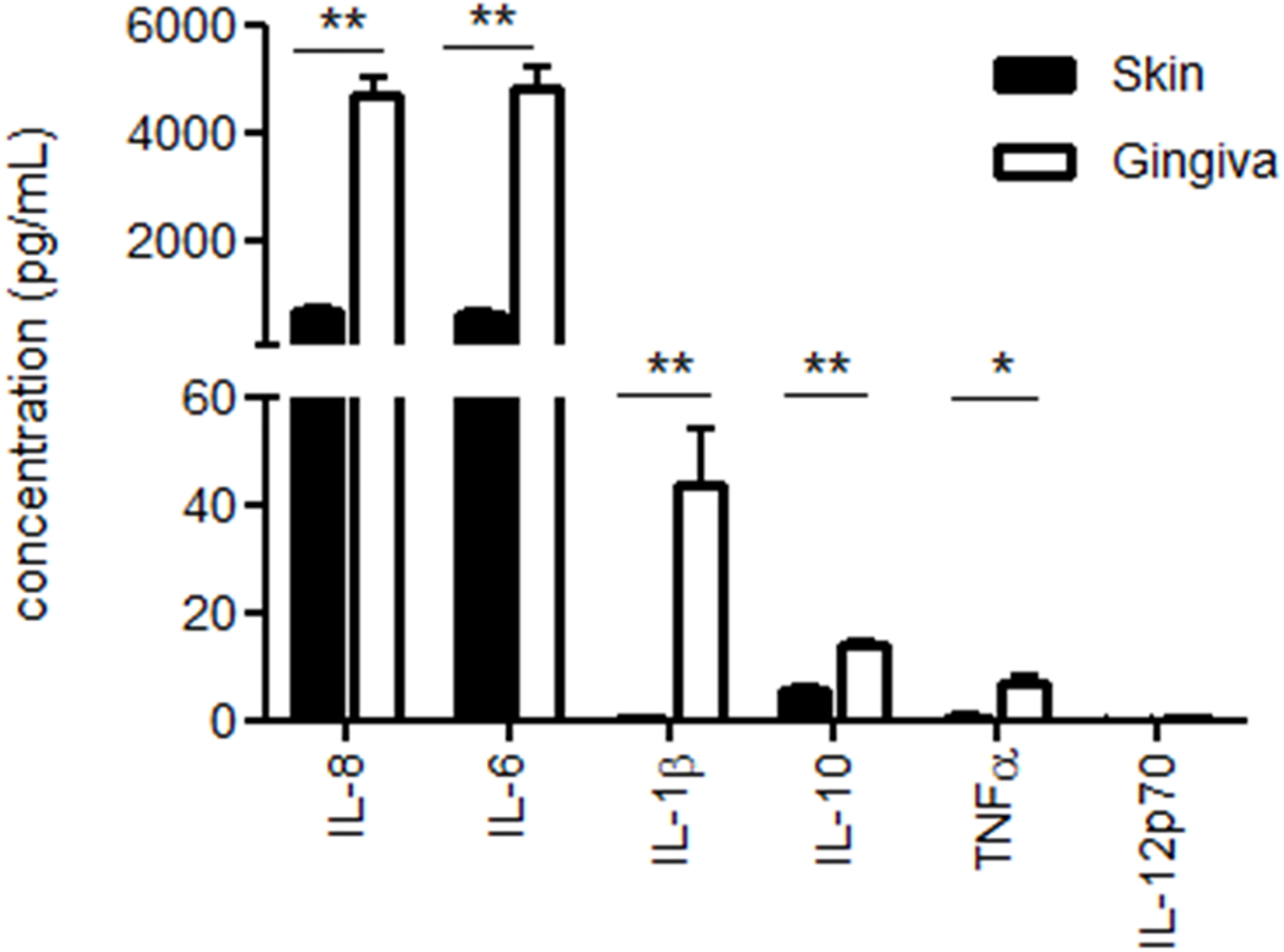
Inflammatory cytokine release profile of skin vs. gingival explants. Shown in pg/ml and measured in supernatants of skin and gingiva explants (6 mm diameter, 3mm depth) after 48 h of culture in 1 ml volume. IL-8, IL-6, IL-1β, IL-10 and TNFα were all significantly higher in the gingiva-conditioned cultures, whereas IL-12p70 levels were below the detection limit for both skin and gingiva (*P<0.05, **P<0.05; n = 3 skin, n = 3 gingiva).

We next tested the conditioned media from 48 h gingival and skin explant cultures for the release of inflammatory cytokines and observed strikingly higher levels of virtually all tested cytokines in the gingival cultures (Fig 4).

**Fig 5:**
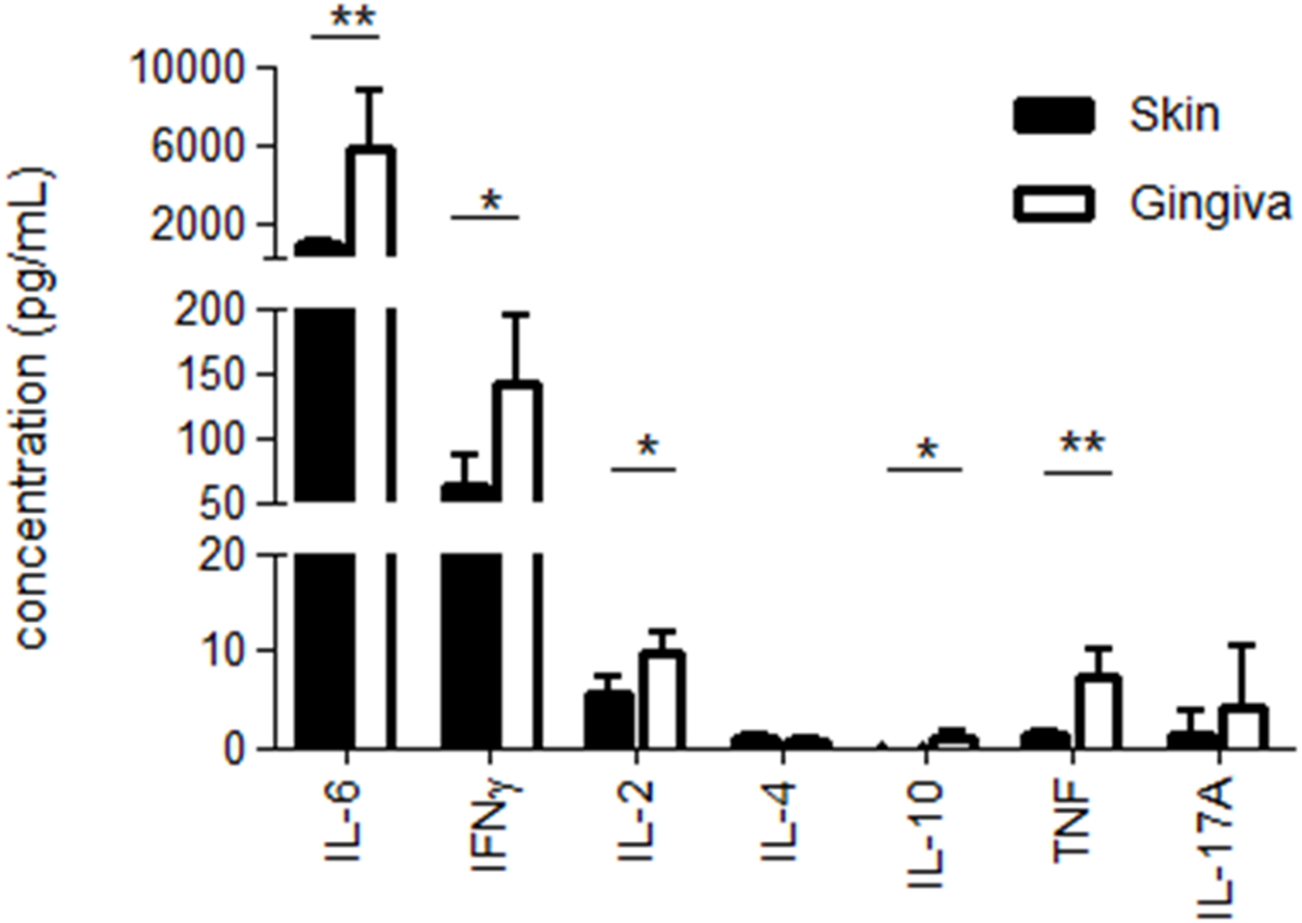
T cell cytokine release in allogeneic mixed leukocyte reactions. T cell cytokine release in allogeneic mixed leukocyte reactions with skin vs. gingiva emigrated dendritic cells (DC) shown in pg/ml and measured after 7 days in supernatants of co-cultures of DC and allogeneic peripheral blood lymphocytes (1:10 ratio). Preferential Th1 skewing by gingiva-emigrated DC was demonstrated, and at superior levels to skin-derived DC (*P<0.05, **P<0.05; n = 3 skin, n = 3 gingiva).

IL-8, IL-6, IL-1β, IL-10 and TNFα were all significantly higher in the gingiva-conditioned cultures, whereas IL-12p70 levels were below the detection limit for both skin and gingiva. To assess and compare the ability of 48 h skin- and gingiva-emigrated DC to prime and skew T cell responses, crawl-out DC were co-cultured with a fixed number of allogeneic peripheral blood lymphocytes (PBL, i.e. monocyte-depleted peripheral blood mononuclear cells; 3,000 DC: 30,000 lymphocytes) for seven days after which supernatants were harvested and the release of Th1-, Th2- and Th17-related cytokines were determined. In addition, a PBL only condition was included as a control sample. As shown in Fig 5, gingiva-derived DC turned out to be more powerful inducers of T cells. Taking IFNγ as an indicator of type-1 T cell priming, gingiva-emigrated DC on average induced 2.3-fold higher T cell reactivity. Whereas IL-6 and IL-10 may in part also derive from DC in the co-cultures, concerted release of significantly higher levels of IFNγ, IL-2 and TNFα, together with a complete failure to release detectable levels of IL-4, point to a preferential Th1 skewing by gingiva-emigrated DC, and at superior levels to skin-derived DC. The allogeneic PBL by themselves did not release any of the measured cytokines at levels surpassing the limit of detection (data not shown).

## Discussion

Traditionally the oral route of antigen delivery is regarded as a sure way to induce immune tolerance. There is however a gap in our knowledge of differences in the phenotype and functionality between DC subsets of human skin and oral mucosa, which in large part will determine the outcome of T cell induction upon antigen exposure. The data provided in this manuscript are a first step towards a more detailed inventory and phenotypic and functional profiling of DC subsets in the oral mucosa, more specifically gingiva, in comparative analyses with human skin DC subsets. The skin is commonly regarded as an attractive gateway for the delivery of (tumor) vaccines whereas the oral mucosa is regarded as a gateway for the delivery of immune modulatory de-sensitization therapies e.g. hypo-sensitization sublingual immunotherapy (SLIT) [12,18–22]. It is known that DC subsets are able to migrate to draining LN, even in the steady state, and so maintain peripheral tolerance. Remarkably we found phenotypically equivalent LC and DC subsets migrating from skin and gingiva explants and in the same frequency distribution. Moreover, LC and interstitial DC subsets displayed a similar distribution between epithelium and dermis/lamina propria with, except for the CD14^+^ subsets, higher densities in the oral mucosa, similarly to earlier reported for oral versus nasal mucosa [23]. As CD14^+^CD163^+^ subsets were previously identified as suppressive with the ability to expand Tregs [6,24,25], one might have expected a predominance of these subsets among gingiva-migrated DC. This however turned out not to be the case. Indeed, cytokine release profiling even pointed to a more pro-inflammatory microenvironment in the gingiva than in skin even though higher levels of the immune suppressive IL-10 were found in gingiva, IL-10 can also be produced in the context of inflammatory conditions as a feedback mechanism. Moreover, a superior ability of gingiva-emigrated DC to prime allogeneic T cells and skew them towards a type-1 functional state was observed.

It is well established that inflammatory responses and allergic reactions can occur in the oral cavity as well as the skin [26,27]. Extensive literature on mucosal tolerization, generally refers to the gut, which has a clear immunosuppressive character compared to the inflammatory properties of the skin. When referring to “oral” tolerance, often “gut” tolerance is actually meant [28–32]. Indeed, oral tolerance induction may be mediated by eventual antigen exposure to mucosa of the lower gastrointestinal tract rather than to oral mucosa. Our findings certainly support this notion.

For oral mucosa, two alternative scenarios may be possible upon antigen exposure: i) tolerance may occur in response to the continuous exposure to antigens derived from commensal bacteria, and other non-pathogenic factors, which otherwise might lead to chronic inflammation [14,33] or ii) an immune response may be induced to eliminate pathogenic or noxious factors (e.g. allergens, pathogenic microbes or toxins). Our results strongly support the latter scenario as a default setting. This would suggest that rather than mediating immune tolerance, like the gut, the oral mucosa rather has immune stimulatory properties more akin to the skin. In line with our findings, Hasséus *et al*. demonstrated that LC in human oral buccal epithelium were more efficient primers of T cells than their counterparts in skin. Additionally, in their study CD83-positive cells were found in higher numbers in oral buccal epithelium than in skin epidermis, thus supporting the finding that oral LC are in a higher steady-state maturation state and have higher T cell stimulating capacity than skin LC [13]. One could argue that like skin, the mouth is a gatekeeper and major barrier to the outside world where strong immune defences should be up at all times, e.g. to keep harmful microbes from reaching the gastrointestinal tract. In contrast, in the gut it may be more important to prevent chronic inflammation, which could prove life threatening, and steady-state antigen exposure there may therefore rather lead to immune tolerance.

Nevertheless, caution is called for and additional studies warranted before definitive conclusions can be reached. Different mucosal surfaces with different histology are present within the oral cavity, which may serve different functions. Very little is known about differences between DC subsets deriving from these different mucosal surfaces and their immune competence. Clearly, additional studies will have to delineate these differences in order to obtain a full picture of the immunological outcome of antigen exposure to the oral mucosa. In addition, DC subsets and the microenvironment of oral mucosa-draining secondary lymphoid organs will have to be taken into account. In terms of determining the cytokine skewing abilities of the skin-and gingiva-derived DC, some caution is warranted in interpreting the MLR data presented in this paper. Since we had depleted monocytes from the allogeneic PBL fraction, rather than using purified naïve CD4^+^ T cells for the co-cultures, there may have been very low numbers of remaining APC (i.e. peripheral blood DC subsets and B cells) that could conceivably have stimulated the lymphocytes in an autologous fashion (a so-called auto-MLR response), resulting in potential T cell skewing and possible cytokine release. However, we found no evidence of this as no cytokine release of any kind was detected in the PBL cultures without added skin- or gingiva-derived DC. Thus, we can conclude that the observed release of effector T cell cytokines was mostly, if not exclusively, effected by the skin- and gingiva-derived DC added to the co-cultures.

A striking difference between the more mature CD1a^+^ DC subsets from gingiva and skin was the expression level of CXCR4: high in skin, absent in the oral mucosa. CXCR4 is a chemokine receptor which has a proven pivotal role in the migration of maturing LC from epidermis to dermis in response to dermal fibroblast-derived CXCL12: a first step en route to the draining lymph nodes [3]. Our findings indicate that gingival LC in contrast migrate to the lamina propria in a CXCR4/CXCL12 independent fashion. In keeping with this, researchers found that CXCL12 was not secreted by gingival fibroblasts, not even after their activation [16], and that in gingival equivalents LC migrated to the lamina propria in a CXCL12 independent manner [34]. There are indications that LC in the oral mucosa may not even have to migrate to lymph nodes in order to direct a T cell immune response [35]. Indeed, it has been reported that oral LC present antigens to T cells in the lamina propria in so-called oral lymphoid foci, indicating that oral LC do not need to travel to nearby draining lymph nodes [35] which would require sequential CXCL12 and CCL19/21 gradients [3,36–40]. DC migration to the lamina propria does not require maturation and could thus potentially contribute to immune tolerance induction, provided an immune tolerant milieu prevails in the lymphoid foci [26,41]. Observations reported by Allam and co-workers indeed support this scenario [12]. It is conceivable that the priming of type-1 T cell mediated immunity may require a higher maturation state of the migrating DC and subsequent T cell induction in draining lymph nodes.

Taken together, our data suggest the oral mucosa to be an attractive site for type-1 T cell immunity induction, possibly even more so than skin. Thus, one might even consider oral immunization strategies to combat cancer or viral infection, as long as subsequent antigen exposure in the gut is prevented (e.g. by the use of aerosol-formulated antigens in the form of an oral spray). Clearly, more in-depth analysis is warranted to delineate the functional contribution of the different DC subsets, migrating from different mucosal compartments within the oral cavity, to the induction of type-1 T cell responses. These studies may be seriously hampered by the scarcity of available healthy oral mucosa tissue samples and may require the development of representative equivalent tissue models, such as the one we recently established for gingiva [16,34]. The DC migration as witnessed in our assays is most likely induced by the tissue damage associated with taking the biopsies/explants, resulting in release of cytokines and damage-associated molecular patterns. As such it resembles allergen-induced migration that may be related to TLR triggering. Assessment of the functional abilities of the different DC subsets will eventually allow for the design of fine-tuned DC-targeted vaccine formulations combined with optimally effective oral adjuvants.

## Supporting information

S1 File Different ways to count LC. Raw data file for Fig 1B.
(XLS)

S2 File IHC cell density comparison between skin and gingiva. Raw data file for Table 2.
(XLS)

S3 File FACS results for crawl out experiments. Raw data file for Figs 2 and 3.
(XLS)

S4 File CBA results. Raw data files for Fig 4.
(XLS)

S5 File CBA results. Raw data files for Fig 5.
(XLS)

S6 File PBL Only CBA results. These results support our findings in Figs 4 and 5.
(XLSX)

## Abbreviations

APC: antigen presenting cell
CBA: Cytometric Bead Assay
CTL: cytotoxic T cell
DAMP: damage associated molecular pattern
DDC: dermal dendritic cell
DC: dendritic cell
DN: double negative
DP: double positive
LC: Langerhans cell
LN: lymph node
MLR: mixed lymphocyte reaction
O/N: over night
PAMP: pathogen associated molecular pattern
PBL: peripheral blood lymphocytes
PBMC: peripheral blood mononuclear cells
RT: room temperature
Treg: regulatory T cell
